# Polymorphic variants at *NDUFC2*, encoding a mitochondrial complex I subunit, associate with cardiac hypertrophy in human hypertension

**DOI:** 10.1186/s10020-023-00701-x

**Published:** 2023-08-09

**Authors:** Giovanna Gallo, Maurizio Forte, Maria Cotugno, Simona Marchitti, Rosita Stanzione, Giuliano Tocci, Franca Bianchi, Silvia Palmerio, Mariarosaria Scioli, Giacomo Frati, Sebastiano Sciarretta, Emanuele Barbato, Massimo Volpe, Speranza Rubattu

**Affiliations:** 1 Department of Clinical and Molecular Medicine, School of Medicine and Psychology, Sapienza University, Rome, Italy; 2 IRCCS Neuromed, Pozzilli (Is), Italy; 3 Department of Medicine, University of Verona School of Medicine, Verona University Hospital Trust, Verona, Italy; 4 Department of Medical-Surgical Sciences and Biotechnologies, Sapienza University of Rome, Latina, Italy; 5 IRCCS S. Raffaele, Rome, Italy

**Keywords:** Hypertension, Cardiac hypertrophy, *NDUFC2*, Mitochondrial dysfunction, Mitochondrial complex I, SIRT3

## Abstract

**Background:**

A dysfunction of NADH dehydrogenase, the mitochondrial Complex I (CI), associated with the development of left ventricular hypertrophy (LVH) in previous experimental studies. A deficiency of Ndufc2 (subunit of CI) impairs CI activity causing severe mitochondrial dysfunction. The T allele at *NDUFC2*/rs11237379 variant associates with reduced gene expression and impaired mitochondrial function. The present study tested the association of both *NDUFC2*/rs11237379 and NDUFC2/rs641836 variants with LVH in hypertensive patients. In vitro studies explored the impact of reduced Ndufc2 expression in isolated cardiomyocytes.

**Methods:**

Two-hundred-forty-six subjects (147 male, 59.7%), with a mean age of 59 ± 15 years, were included for the genetic association analysis. *Ndufc2* silencing was performed in both H9c2 and rat primary cardiomyocytes to explore the hypertrophy development and the underlying signaling pathway.

**Results:**

The TT genotype at *NDUFC2/*rs11237379 associated with significantly reduced gene expression. Multivariate analysis revealed that patients carrying this genotype showed significant differences for septal thickness (p = 0.07), posterior wall thickness (p = 0.008), RWT (p = 0.021), LV mass/BSA (p = 0.03), compared to subjects carrying either CC or CT genotypes. Patients carrying the A allele at *NDUFC2/*rs641836 showed significant differences for septal thickness (p = 0.017), posterior wall thickness (p = 0.011), LV mass (p = 0.003), LV mass/BSA (p = 0.002) and LV mass/height^2.7^(p = 0.010) after adjustment for covariates. *In-vitro*, the Ndufc2 deficiency-dependent mitochondrial dysfunction caused cardiomyocyte hypertrophy, pointing to SIRT3-AMPK-AKT-MnSOD as a major underlying signaling pathway.

**Conclusions:**

We demonstrated for the first time a significant association of *NDUFC2* variants with LVH in human hypertension and highlight a key role of Ndufc2 deficiency-dependent CI mitochondrial dysfunction on increased susceptibility to cardiac hypertrophy development.

**Supplementary Information:**

The online version contains supplementary material available at 10.1186/s10020-023-00701-x.

## Introduction

Left ventricular hypertrophy (LVH) is an initially adaptive process occurring when the heart puts in place pathophysiological mechanisms to compensate an increased hemodynamic burden (Lorell & Carabello 2000, Nakamura & Sadoshima 2018). Chronic hypertrophy leads to increased wall stress and myocardial stiffness (Kuwahara et al. 2004; van Heerebeek et al. 2012). Persistent cardiac hypertrophy is associated with deranged energy metabolism. In such a context, mitochondria play a fundamental role in the production of the energy necessary for an adequate contraction and relaxation of cardiomyocytes to meet the workload demand (Rosca et al. 2013). A common feature of mitochondrial dysfunction in the heart is the disruption of mitochondrial complex I (CI), known as NADH: ubiquinone oxidoreductase, caused either by mutations in the genes responsible of a fully functional complex or by cumulative damage to the complex itself (Peoples et al. 2019). A dysfunctional CI results in an increased mitochondrial reactive oxygen species (ROS) accumulation and deleterious cellular effects which may contribute to the development of cardiac hypertrophy (Forte et al. 2019). Previous studies showed that a deficiency of CI subunits (Ndufs1 or Ndufc4) led to defective activity of the electron transport chain (ETC) with decreased ATP production, impaired mitochondrial dynamics and, within the heart, development of LVH (Chouchani et al. 2014; Ni et al. 2019; Zou et al. 2021). A role of Sirtuin 3 (SIRT3) was suggested in this context.

The Ndufc2 subunit is fundamental for the assembly and activity of CI (Gershoni et al. 2014). Ndufc2 deficiency leads to mitochondrial dysfunction and increased oxidative stress at the cellular level (Raffa et al. 2017; Rubattu et al. 2016). *In-vivo*, a heterozygous Ndufc2_knock out (KO) rat model, obtained from the stroke-resistant spontaneously hypertensive rat strain (SHRSR), once fed with a high-salt/low potassium Japanese style diet, developed renal damage and stroke, resembling the phenotype of the stroke-prone SHR (SHRSP). The effects of Ndufc2 deficiency on oxidative stress and mitochondrial damage were confirmed in mitochondria from peripheral blood mononuclear cells (PBMCs) of human subjects carrying the T allele at rs11237379/*NDUFC2*, associated with decreased *NDUFC*2 expression and mitochondrial dysfunction (Raffa et al. 2017). An increased predisposition to cardiovascular events was observed in these subjects (Gallo et al. 2022; Rubattu et al. 2016). The pathogenic relevance of the rs11237379 variant was enhanced by the concomitant presence of a second allele variant at rs641836*/NDUFC2* (Rubattu et al. 2016).

The impact of Ndufc2 deficiency on cardiac hypertrophy development has not been investigated yet.

In our study we first examined the putative association of the two known *NDUFC2* polymorphic variants with LVH in a cohort of Italian hypertensive patients. As a second step, we explored the effect of reduced Ndufc2 expression on hypertrophy development in isolated cardiomyocytes and the underlying molecular mechanisms.

## Methods

### Human study

Two hundred and forty-six consecutive unrelated Caucasian adults who were referred for hypertension management to the Department of Cardiology, Sapienza University, Sant’Andrea Hospital, Rome, were enrolled in this study from October 2018 to December 2021.

Hypertension was diagnosed based on the presence of office systolic blood pressure (SBP) values ≥ 140 mmHg and/or diastolic BP (DBP) values ≥ 90 mmHg (average of 3 repeated measurements made by the same physician with an oscillometric automatic sphygmomanometer)(Williams et al. 2018). Also subjects who self-reported treatment with antihypertensive drugs, namely ACE inhibitors (ACEi), angiotensin receptor blockers (ARB), calcium channel blockers (CCB), thiazide/thiazide-like diuretics, loop diuretics, mineralocorticoid receptor antagonists (MRA), beta-blockers and alpha-blockers, were considered hypertensives. From an initial sample size of 2.125 hypertensive patients, we excluded from the analysis those with clinical conditions potentially causing LVH or representing a bias in the interpretation of the performed echocardiograms: cardiomyopathies (n = 45), moderate-to-severe valvular heart diseases (n = 247), significant peripheral vascular disease (n = 131), coronary artery disease (n = 440), abnormal LV function or wall motion abnormalities (n = 224), pericardial disease (n = 32), obesity (body mass index [BMI] ≥ 30 Kg/m^2^) (n = 184), severe obstructive/restrictive lung disease (n = 97), obstructive sleep apnea (n = 105), resistant hypertension (n = 147), secondary hypertension (n = 91), moderate or severe chronic kidney disease (estimated glomerular filtration rate > 30–59 mL/min/1.73 m^2^ and < 30 mL/min/1.73 m^2^, respectively) (n = 110). Twenty-six subjects refused to sign the informed consent to the study. The presence of hypercholesterolemia, diabetes mellitus, smoking habit and pharmacological treatment was recorded. Physical examination, BP measurements, a 12-lead ECG were performed in all patients. ECG LVH was defined based on the 2018 European Society of Cardiology/European Society of Hypertension (ESC/ESH) Guidelines(Williams et al. 2018). Since ECG is not a sensitive method for detecting LVH, all patients underwent mono-dimensional and bi-dimensional transthoracic echocardiography. LV internal diameters, septal thickness, and posterior wall thickness were measured according to the guidelines of the American Society of Echocardiography and European Association of Cardiovascular Imaging (Lang et al. 2015). The LV mass was calculated at the end diastole by applying the Devereux correction to the American Society of Echocardiography cube LV mass formula. The relative wall thickening (RWT) was calculated as: 2 * posterior wall thickness divided by LV diastolic diameter or, septal wall thickness + posterior wall thickness divided by LV diastolic diameter. We followed the 2018 ESC/ESH Guidelines (Williams et al. 2018) to define LVH by echocardiography.

### Genomic DNA extraction and genotyping

Genomic DNA was extracted by a commercially available kit (Qiagen, Milan, Italy). The T/C *NDUFC2/*rs11237379 and G/A *NDUFC2/*rs641836 polymorphic variants were characterized as previously reported (Rubattu et al. 2016). The real-time polymerase chain reaction (RTqPCR) was performed by a TaqMan technology assay (Life Technologies, Carlsbad, CA, USA) using the ViiA 7 Real-Time PCR System (Applied Biosystem, Foster City, CA, USA).

### Peripheral blood mononuclear cells (PBMCs) isolation, ***NDUFC2*** and ***SIRT3*** expression analysis

A total of 10 hypertensive patients carrying either TT or CC/rs11237379 genotype (n = 5 for each genotype in each group), while being wild type at rs641836, were recruited for the isolation of PBMCs and assessment of the *NDUFC2* and *SIRT3* expression level.

PBMCs were isolated by the ficoll procedure, as previously described (Raffa et al. 2019). Total RNA was extracted by TRIZOL, and 1000 ng were retrotranscribed into cDNA by the Superscript VILO (Invitrogen, 11756050, Waltham, MA, US). RTqPCR was performed with SsoAdvance Universal SYBR Green supermix (Bio-Rad, 1725270) on a Real-Time PCR system (CFX96; Bio-Rad, Milan, Italy), according to the manufacturer’s protocols. The following primers were used for the RTqPCR: *SIRT3* For: 5’-CCGGATCAGCACACCCAGT-3’, Rev: 5’-CTCGGGGTACGGGAGATCGT-3’; *NDUFC2* For: 5’-CCTGTATGCTGTGAGGGACC-3’, Rev: 5’-CGACGTTCAGCTCCACAACA-3’; *β-ACTIN* For: 5’-ATCACCATTGGCAATGAGCG-3’, 5’-TTGAAGGTAGTTTCGTGGAT-3’.

### Cell cultures

In vitro experiments were performed both in H9c2 myoblasts (ATCC®, CRL-1446™) and in neonatal rat ventricular cardiomyocytes (CMs). H9c2 were cultured in DMEM high-glucose medium (Gibco) supplemented with 10% FBS and antibiotics at 37 °C and 5% CO_2_. H9c2 were used between passages 1–9. CMs were isolated from 1 to 2-day-old neonatal Wistar Kyoto rats by the Miltenyi technology (Miltenyi Biotec, 130-098-373, Bergisch Gladbach, Germany) and cultivated as previously described (Forte et al. 2022).

### Ndufc2 gene silencing and NMN treatment

Gene silencing for *Ndufc2* in H9c2 and in CMs (*Ndufc2*-KD) was performed by using a rat-specific commercially available siRNA (Ndufc2 Silencer Select Pre-designed siRNA, 4390771, Ambion, Waltham, MA, US) and following the manufacturer’s protocol. siRNA was diluted in OPTIMEM-reduced serum medium (Thermo Fisher Scientific, 31985062, Waltham, MA, US) with the transfection agent Lipofectamine RNAi max (Thermo Fisher Scientific, 13778150). After 5 hours, OPTIMEM was replaced with complete medium. The evaluation of cell hypertrophy, mitochondrial function and biomolecular analyses were performed after 48 hours. For rescue experiments, CMs were treated with nicotinamide mononucleotide (NMN) (N3501, Sigma Aldrich, Milan, Italy) 1 µM for 48 hours. Control cells were incubated with Lipofectamine RNAi max and a scramble siRNA (Silencer™ Select Negative Control No. 1 siRNA, Thermo Fisher Scientific, 4390843), diluted in OPTIMEM. The efficiency of gene silencing was confirmed by RTqPCR (Additional file, Fig. S1). RTqPCR was performed as described above, using glyceraldehyde-3-phosphate dehydrogenase (*Gapdh*) as housekeeping gene. The primers used were the following: *Ndufc2* For: 5’-GCTGTGAGGGACCATGA-3’, Rev: 5’-GGCTCAAGAATTTCAGCATAAG-3’. *Gapdh* For: 5’-AACGACCCCTTCATTGACCTC-3’, Rev: 5’-CCTTGACTGTGCCGTTGAACT-3’.

### Cell hypertrophy assessment

Hypertrophy was assessed by immunofluorescence for β-actin. Cells were fixed with PFA 4%, washed with PBS 1X and incubated overnight at 4°C with the primary antibody (sc-69879 Santa Cruz Biotechnology, Dallas, Texas). Afterword, cells were incubated with a FITC-conjugated secondary antibody for 1 hour at room temperature. Cell nuclei were stained with Hoechst (62249, Thermo Fisher). Images were acquired with a fluorescence microscope and cell size was quantified by ImageJ (National Institutes of Health, USA). Cell hypertrophy was also investigated by analyzing atrial natriuretic peptide (ANP) and β- heavy chain cardiac myosin (MHC) by RTqPCR and by western blot. Primers used for *Nppa* and β-*Mhc* were the following: β-*Nppa* For: 5’-TTCAAGAACCTGCTAGACCAC-3’, Rev: 5’-CCTCAGAGAGGGAGCTAAGT-3’ *Mhc* For: 5’-CCTCCCAAGTTCGACAAGAT-3’, Rev: 5’-AGGCCTGAGTAGGTGTAGAT-3’. *Gapdh* was used as houseeking gene.

### Mitochondrial function determination

NAD^+^: NADH levels were measured in cells by using a commercially available colorimetric kit (ab65348, Abcam, Cambridge, UK) following the manufacturer’s instructions.

Mitochondrial membrane potential (ΔΨm) in primary CMs was assessed by fluorescence microscopy through the 5, 50,6,60-tetrachloro-1,10,3,30-tetraethylbenzimidazolylcarbocyanine iodide probe (JC-1 Dye; Molecular Probes, Invitrogen, T3168) following the manufacturer’s instructions. Images were acquired in red (em 590 nm) and green (em 525 nm) field and the red/green (R: G) ratio was calculated by ImageJ.

### Oxidative stress determination

Intracellular Reactive Oxygen Species (ROS) generation following *Ndufc2* silencing were determined in both H9c2 and CMs by the OxiSelectTM Intracellular ROS Assay Kit (Cell Biolabs, Inc., San Diego, CA, US) following the manufacturer’s instructions. Briefly, 1 × 10^4^ cells were plated in 96 Well Black/Clear Bottom Plate (Sarstedt, Nümbrecht, Germany); they were silenced and incubated with the cell-permeable fluorogenic probe 2’, 7’-Dichlorodihydrofluorescin diacetate (DCFH-DA). The rescue with NMN was evaluated in primary CMs. Values of fluorescence were read with a microplate reader.

### Western blot

Proteins were extracted with lysis buffer and western blot analysis was performed as previously reported (Forte et al. 2022). The following primary antibodies were used: anti-SIRT3 (5490S, Cell Signaling Technology, Danvers, MA, US), anti-protein kinase B (AKT) (9712, Cell Signaling Technology), anti-phosphoAKT (Ser-473, 12694, Cell Signaling Technology), anti-5’ AMP-activated protein kinase (AMPK) (2603, Cell Signaling Technology), anti-phosphoAMPK (Thr172, 2535, Cell Signaling Technology), anti-MHC (ab50967, abcam), anti-ANP (ab225844, abcam), anti-GAPDH (97,166, Cell Signaling Technology), anti-Vinculin (sc-73,614, Santa Cruz Biotechnology, Dallas, TX, US), anti-β-ACTIN (sc69879, Santa Cruz Biotechnology), anti-Manganese Superoxide Dismutase (MnSOD) (NBP2-20535, Novus Biological, Centennial, CO, US). Secondary antibodies were anti-rabbit (AP132P, Millipore, Burlington, MA, US) and anti-mouse (Millipore, 401,215).

### Statistical analysis

Statistical analysis was performed using the SPSS version 25 (SPSS Inc., Chicago, Illinois). In the human study categorical variables are expressed as frequencies and percentages. Continuous data are given as means ± SD. Differences between groups were analyzed either by t test or 1-way analysis of variance (ANOVA) if groups were more than two. The Bonferroni post-hoc least significant difference test was performed to complete the analysis for multiple comparisons. A covaried 1-way ANOVA was performed considering each cardiac parameter as a dependent variable, considering age, gender, BMI, office BP, antihypertensive treatment with a combination of two or more drugs and the number of BP-lowering agents as covariates, and considering the polymorphism showing significant differences in the analyses among groups as an independent value. A multiple logistic regression analysis was used to estimate odds ratios (ORs) and 95% confidence intervals for risk of LVH under the assumption of an additive, as well as a dominant and a recessive effect of each allele. To evaluate whether polymorphisms were independently associated with LVH, multiple logistic regression analyses were adjusted for the abovementioned covariates.

Deviation from the Hardy-Weinberg equilibrium was analyzed with the chi-square test. A value of P < 0.05 was chosen as the cut-off level for statistical significance. Results of the in vitro studies are provided as means ± S.E.M. Differences between two groups (*Ndufc2*-KD vs. CTR) were analyzed by Student t-test whereas one-ANOVA followed by Bonferroni post-hoc test was used for differences between multiple groups (CTR, *Ndufc2*-KD, NMN). Graphs and statistical analyses of in vitro studies were performed by Graph Pad Prism (GraphPad Software, Inc. La Jolla, CA, USA) and statistical significance was set at P < 0.05 level. The n of experiment is reported in the figure legends.

## Results

### Human study

A total of 246 hypertensive subjects (144 male, 58.6%) with a mean age of 59 ± 15 years were included. The main anthropometric, clinical, echocardiographic, and pharmacological variables are reported in Table 1. Seventy-nine patients (32%) presented LVH according to the 2018 European Guidelines echocardiographic criteria. Apart from the echocardiographic parameters, hypertensive patients with and without LVH had comparable clinical characteristics (Table 2). Regarding the ongoing treatments, patients with LVH received more often ACEi. The alleles and genotypes distribution of the two gene variants are reported in Table 3.

**Table 1:** Characteristics of the overall population (n = 246)

General data	
Age, years	59 ± 15
Male sex, n (%)	144 (58.6)
BMI, Kg/m^2^	25.7 ± 3.6
Diabetes, n (%)	41 (16.7)
Dyslipidaemia, n (%)	97 (40)
Clinic SBP, mmHg	145.6 ± 16.9
Clinic DBP, mmHg	87.1 ± 9.8
24 h mean SBP, mmHg	129.1 ± 13.1
24 h mean DBP, mmHg	78.1 ± 8.9
**Echocardiographic data**	
Septal thickness, mm	10.1 ± 1.6
LVPW, mm	9.8 ± 1.6
RWT	0.41 ± 0.06
LVEDd, mm	48.6 ± 4.1
LV mass, g	172.1 ± 47.9
LV mass/BSA, g/m^2^	93.2 ± 23.8
LV mass/h^2,7^, g/m^2,7^	39.6 ± 13.1
LA vol/h^2^, ml/m^2^	15.3 ± 3.4
LVEF, %	67.5 ± 9.7
**Pharmacological therapy**	
ACEi, n (%)	46 (18.6)
ARB, n (%)	111 (45)
Calcium channel blockers, n (%)	93 (37.7)
Thiazide diuretics, n (%)	64 (26)
Beta-blockers, n (%)	55 (22.5)
MRA, n (%)	11 (4.3)
Combination therapy, n (%)	126 (51)
2 drugs, n (%)	88 (35.6)
3 drugs, n (%)	27 (11)
4 drugs, n (%)	11 (4.5)

ACEi, angiotensin converting enzyme inhibitors; ARB, angiotensin receptor blockers; BMI, body mass index; DBP, diastolic blood pressure; LA, left atrium; LV, left ventricle; LVEDd, left ventricular end-diastolic diameter; LVEF, left ventricular ejection fraction; LVPW, left ventricular posterior wall; MRA, mineracolorticoid receptor antagonists; RWT, relative wall thickening; SBP, systolic blood pressure

**Table 2:** Characteristics of the population according to LVH

General data	Without LVH (n = 167)	With LVH (n = 79)	p-value
Age, years	58 ± 14	60 ± 15	NS
Male sex, n (%)	91 (56)	45(60)	NS
BMI, Kg/m^2^	26.1 ± 3.6	25.2 ± 3.3	NS
Diabetes, n (%)	29 (18)	12 (16)	NS
Dyslipidaemia, n (%)	64 (38)	33 (41)	NS
Clinic SBP, mmHg	144 ± 17	146 ± 18	NS
Clinic DBP, mmHg	87 ± 9	87 ± 10	NS
24 h mean SBP, mmHg	130 ± 14	131 ± 12	NS
24 h mean DBP, mmHg	79 ± 9	77 ± 9	NS
**Echocardiographic data**			
IVSd, mm	9.5 ± 1.1	11.2 ± 1.6	< 0.001
LVPWd, m	9 ± 1.2	11.1 ± 1.5	< 0.001
RWT	0.38 ± 0.05	0.45 ± 0.07	< 0.001
LVEDd, mm	48 ± 4	50 ± 4	0.011
LV mass, g	150.6 ± 32.7	206.9 ± 48.3	< 0.001
LV mass/BSA, g/m^2^	80.6 ± 13.4	113.8 ± 22.9	< 0.001
LV mass/h^2,7^, g/m^2,7^	33.2 ± 10	50.5 ± 10	< 0.001
LA vol/h2, ml/m2	14.0 ± 2.0	18.6 ± 4.1	< 0.001
LVEF, %	68 ± 10	69 ± 10	NS
**Pharmacological therapy**			
ACEi, n (%)	35 (21)	10 (13)	0.032
ARB, n (%)	67 (43)	38 (48)	NS
Calcium channel blockers, n (%)	60 (36)	31 (39)	NS
HCTZ, n (%)	45 (27)	18 (23)	NS
Beta-blockers, n (%)	40 (24)	15 (19)	NS
MRA, n (%)	9 (5)	2 (3)	NS
Combination therapy, n (%)	88 (53)	38 (48)	NS

For abbreviations see Table 1

**Table 3:** Genotype and allele frequencies distribution for rs641836 and rs11237379 NDUFC2 polymorphisms in hypertensive patients with and without LVH

SNP	Patients without LVH (n = 167) n (%)	Patients with LVH (n = 79) n (%)	p-value
rs641836			
GG	114 (68.2)	39 (49.4)	0.004
GA	50 (29.9)	34 (43)	0.047
AA	3 (1.8)	6 (7.5)	0.034
A frequency	0.167	0.291	0.007
H-W	0.47	0.12	
**rs11237379**			
TT	43 (25.7)	32 (40.5)	0.017
TC	96 (57.4)	34 (43)	0.019
CC	28 (16.7)	13 (16.4)	0.513
T frequency	0.545	0.62	0.048
H-W	2.56	0.69	

H-W, Hardy Weinberg; LVH, left ventricular hypertrophy; SNP, single nucleotide polymorphism

The Hardy-Weinberg equilibrium was respected for both markers as documented by chi-square significances.

#### NDUFC2/rs641836 and LVH

Among the whole sample, 152 patients were homozygous for the G allele, 85 patients were heterozygous, and 9 patients were homozygous for the A allele.

The association analysis with the echocardiographic parameters showed that hypertensive patients carrying the AA genotype had a significantly increased septal thickness, posterior wall thickness, RWT, LV mass, LV mass/BSA, LV mass/height^2.7^ and LA volume/height^2^ compared to subjects carrying either the AG or the GG genotype (Table 4).

**Table 4:** One-way ANOVA adjusted for the following covariates: age, gender, BMI, BP, combination therapy, number of antihypertensive drugs with the *NDUFC*2/rs641836 polymorphism as independent variable

Variables	GG n = 152	GA n = 85	AA n = 9	*NDUFC2* rs641836 p-value; (R^2^)	Total model p-value; (R^2^)
Septal thickness (mm)	9.7 ± 1.6 †#	10.4 ± 1.4 *†	12.4 ± 1.8 *#	0.001; (25.0%)	0.002; (17.9%)
LVPW (mm)	9.2 ± 1.5 †#	10.2 ± 1.2 *†	12.0 ± 2.0 *#	0.001; (32.9%)	0.003; (26.5%)
RWT	0.39 ± 0.06 †#	0.42 ± 0.05 *†	0.51 ± 0.09 *#	0.001; (17.5%)	0.036; (9.5%)
LV mass (g)	158.0 ± 42.9 †#	187.0 ± 47.1 *†	229.2 ± 62.7 *#	0.001; (36.2%)	0.004; (30.1%)
LV mass/BSA (g/m^2^)	85.1 ± 20.1 †#	101.5 ± 23.4 *†	128.0 ± 32.2 *#	0.001; (32.0%)	0.002; (25.4%)
LV mass/h^2.7^ (g/m^2.7^)	36.5 ± 11.9 †#	42.4 ± 13.8 *†	54.5 ± 9.1 *#	0.003; (22.3%)	0.011; (15.1%)
LA vol/h^2^ (ml/m2)	14.8 ± 3.4 †	15.1 ± 2.2 †	21.9 ± 3.5 *#	0.008; (11.4%)	0.06; (3.8%)

*<0.05 vs. GG; # <0.05 vs. GA; †<0.05 vs. AA

The column titled *NDUFC2* rs641836 reports the significance (p-value) and amount of variability (R^2^) explained by the effect of the Ndufc2 polymorphism independently of all covariates. The column titled Total Model reports the significance (p-value) and amount of variability (R^2^) explained by the complete analysis. For abbreviations see Table 1

To better dissect out the genetic effect, a covariate ANOVA was performed for each cardiac variable considering age, gender, BMI, office BP, antihypertensive treatment with a combination of 2 or more drugs and the number of BP-lowering agents as covariates. The results were significant for septal thickness, posterior wall thickness, RWT, LV mass, LV mass/BSA and LV mass/height^2.7^ as shown in Table 4 (column titled “Total Model”).

Due to the small number of subjects carrying homozygosity for the A allele, all individuals carrying the A allele were combined to confirm the results. Subjects carrying the A allele (n = 94) had a significant increase in septal thickness, posterior wall thickness, RWT, LV mass, LV mass/BSA and LV mass/height^2^ compared to GG homozygotes (Table 5). The significance was maintained after the adjustment for the abovementioned covariates (column titled “Total Model”).

**Table 5:** Echocardiographic variables according to the carrier status of the mutant A allele at *NDUFC*2/rs641836.

Variables	GG n = 152	GA + AA n = 94	*NDUFC2* rs641836 p-value; (R^2^)	Total model p-value; (R^2^)
Septal thickness (mm)	9.7 ± 1.6	10.6 ± 1.6	0.001; (17.8%)	0.017; (11.0%)
LVPW (mm)	9.2 ± 1.5	10.4 ± 1.4	0.001; (26.8%)	0.011; (20.8%)
RWT	0.40 ± 0.06	0.43 ± 0.06	0.005; (9.8%)	0.257; (2.3%)
LV mass (g)	158.1 ± 42.9	191.1 ± 49.7	0.001; (32.3%)	0.003; (26.7%)
LV mass/BSA (g/m^2^)	85.0 ± 21.4	104.0 ± 25.2	0.001; (26.8%)	0.002; (20.7%)
LV mass/h^2.7^ (g/m^2.7^)	36.7 ± 11.9	43.5 ± 13.8	0.002; (18.7%)	0.010; (12.2%)
LA vol/h^2^ (ml/m^2^)	14.8 ± 3.4	15.6 ± 2.8	0.062 (6.8%)	0.071 (4.7)

Each row represents a covaried 1-way analysis of variance of 1 individual cardiac parameter, considered as the dependent variable. The column titled *NDUFC2* rs641836 reports the significance (p-value) and amount of variability (R^2^) explained by the effect of the carrier status of the mutant A allele of *NDUFC2* rs641836 independently of all covariates (age, gender, BMI, BP, combination therapy, number of antihypertensive drugs). The column titled Total Model reports the significance (p-value) and amount of variability (R^2^) explained by the complete analysis

#### NDUFC2/rs11237379 and LVH

Among the whole sample, 40 patients were homozygous for the C allele, 131 patients were heterozygous, and 75 were homozygous for the T allele.

The association analysis with the echocardiographic parameters showed that hypertensive patients carrying the TT genotype had a significant increase of septal thickness, posterior wall thickness, RWT, LV mass/BSA and LV mass/height^2.7^ (Table 6). These results were confirmed by the covariate ANOVA (column titled “Total Model”).

**Table 6:** One-way ANOVA adjusted for the following covariates: age, gender, BMI, BP, combination therapy, number of antihypertensive drugs with the *NDUFC2/*rs11237379 polymorphism as independent variable

Variables	CC N = 40	CT N = 131	TT N = 75	*NDUFC2* rs11237379 p-value; (R^2^)	Total model p-value; (R^2^)
Septal thickness (mm)	9.7 ± 1.5 †	10.0 ± 1.7	10.7 ± 1.6 *	0.001; (21.4%)	0.007; (13.9%)
LVPW (mm)	9.3 ± 1.4 †	9.5 ± 1.5	10.4 ± 1.6 *	0.003; (21.5%)	0.008; (14.0%)
RWT	0.39 ± 0.05 †	0.41 ± 0.06	0.44 ± 0.07 *	0.01; (19.0%)	0.021; (11.2%)
LV mass (g)	164.4 ± 48.4	165.0 ± 49.3	183.0 ± 45.4	0.065; (5.8%)	0.074; (5.1%)
LV mass/BSA (g/m^2^)	87.5 ± 25.1 †	88.1 ± 23.3 †	101.1 ± 24.7 *#	0.012; (21.0%)	0.03; (13.4%)
LV mass/h^2.7^ (g/m^2.7^)	36.7 ± 12.2 †	40.0 ± 12.3	42.9 ± 14.1 *	0.033 (15.9%)	0.054 (7.9%)
LA vol/h^2^ (ml/m^2^)	13.8 ± 1.8	14.7 ± 3.2	16.3 ± 3.7	0.078 (2.3%)	0.081 (1.7%)

*<0.05 vs. CC; # <0.05 vs. CT; †<0.05 vs. TT

The column titled *NDUFC2* rs11237379 reports the significance (p-value) and amount of variability (R^2^) explained by the effect of the *NDUFC*2 polymorphism independently of all covariates. The column titled Total Model reports the significance (p-value) and amount of variability (R^2^) explained by the complete analysis. For abbreviations see Table 1

#### Combined analysis of NDUFC2/ rs641836/rs11237379 and LVH

Subjects with the combined presence of A rs641836 allele and TT/rs11237379 genotype had a significant increase in septal thickness, posterior wall thickness, RWT, LV mass, LV mass/BSA and LV mass/height2 compared to those carrying the GG/ rs641836 and CC/rs11237379 genotypes. This evidence suggests the existence of an additive effect of the two *NDUFC2* markers (Table 7).

**Table 7:** Echocardiographic variables according to the combination of *NDUFC*2/rs641836 and *NDUFC2*/rs1123737 genotypes

Variables	A rs641836 allele and TT/ rs11237379 genotype (n = 43)	GG/rs641836 and CC/rs11237379 genotype (n = 40)	*NDUFC*2 rs641836/ rs1123737 genotype p-value; (R^2^)	Total model p-value; (R^2^)
Septal thickness (mm)	11.1 ± 1.7	9.7 ± 1.5	0.001; (23.1%)	0.016; (14.3%)
LVPW (mm)	10.8 ± 1.6	9.3 ± 1.4	0.001; (29.7%)	0.031; (22.5%)
RWT	0.46 ± 0.07	0.39 ± 0.05	0.004; (25.1%)	0.008; (16.9%)
LV mass (g)	191.5 ± 49.4	164.4 ± 48.4	0.002; (24.2%)	0.01; (16.3%)
LV mass/BSA (g/m^2^)	105.6 ± 27.1	87.5 ± 25.1	0.001; (24.5%)	0.009; (16.4%)
LV mass/h^2.7^ (g/m^2.7^)	44.3 ± 14.5	36.7 ± 12.2	0.003; (21.2%)	0.027; (12.6%)
LA vol/h^2^ (ml/m^2^)	16.5 ± 3.8	13.8 ± 1.8	0.176; (2.8%)	0.231 (1.7%)

Due to the small number of subjects carrying homozygosity for the A allele at *NDUFC*2 rs641836, all individuals carrying the A allele were combined. The column titled *NDUFC2* rs641836/ rs1123737 genotype reports the significance (p-value) and amount of variability (R^2^) explained by the effect of the combination of different genotypes at *NDUFC2* rs641836 and rs1123737 independently of all covariates. The column titled Total Model reports the significance (p-value) and amount of variability (R^2^) explained by the complete analysis. For abbreviations see Table 1

Multivariable logistic regression analysis, assuming the development of LVH as the dependent variable and both SNPs with the above-mentioned covariates as independent variables, demonstrated that the carrier status of both A allele at rs641836 and TT genotype at rs11237379 was associated with an increased risk of LVH (OR = 2.14 [95% confidence interval, 1.21–3.76]; P = 0.008 and OR = 2.046 [95% confidence interval, 1.14–3.67]; P = 0.017, respectively). (Table 7).

### In vitro studies

#### Ndufc2 downregulation leads to cell hypertrophy in cardiomyocytes

**Fig. 1:**
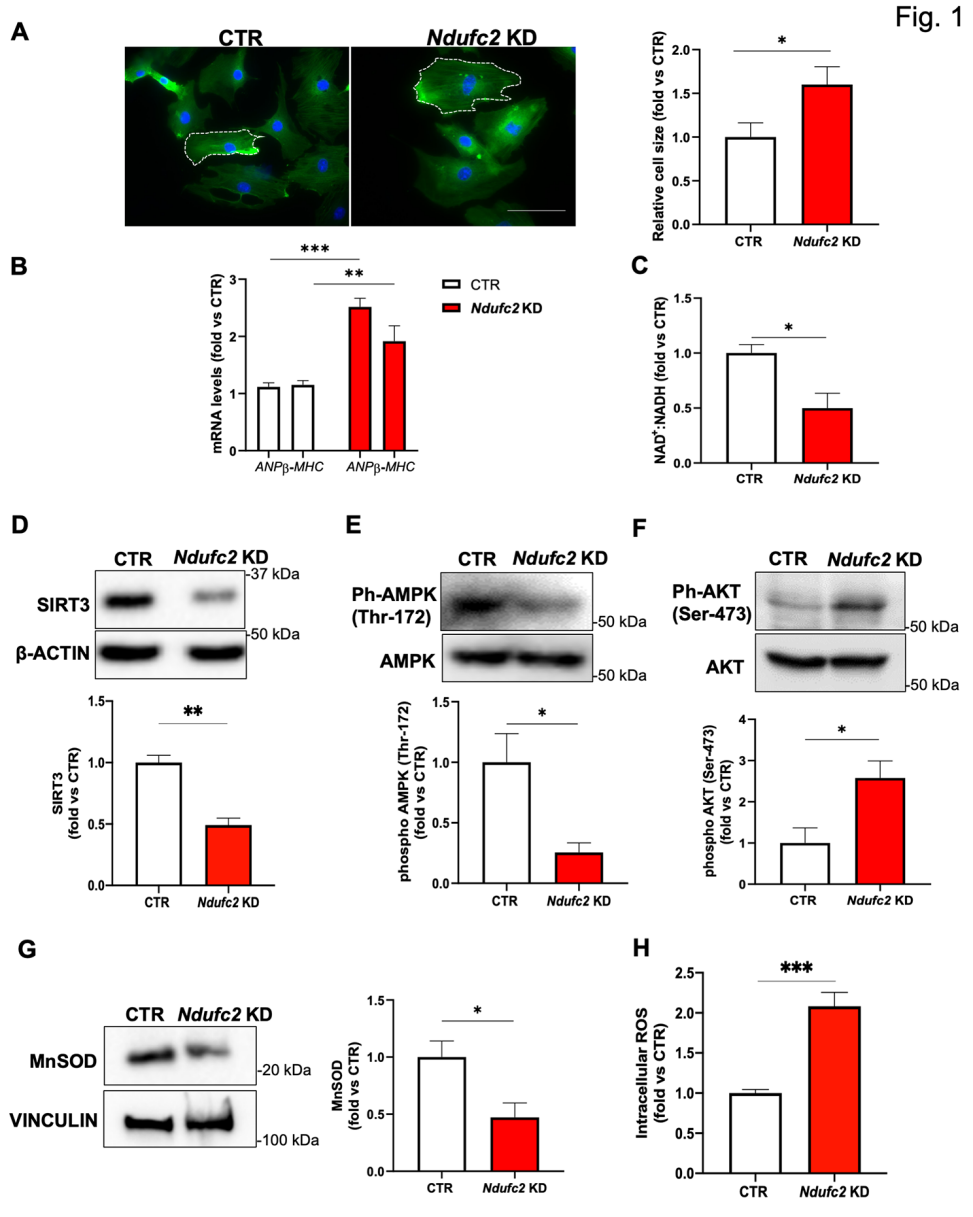
*Ndufc2* silencing leads to cardiac hypertrophy and impairs NAD + metabolism and SIRT3 downstream pathway in H9c2 cells. H9c2 cells were silenced for *Ndufc2* (*Ndufc2*-KD) for 48 h. **A.** Representative images of β-actin fluorescence and relative quantification are shown. **B.** Relative quantification by RTqPCR of *Nppa* and β-*Mhc* mRNA levels. **C.** NAD+:NADH ratio. **D.** Representative western blot for SIRT3 and corresponding quantification. **E.** Representative western blot for phospho-AMPK (Thr-172) and corresponding quantification. **F.** Representative western blot for phospho-AKT (Ser-473) and corresponding quantification. **G**. Representative western blot for MnSOD and corresponding quantification. **H.** Determination of intracellular reactive oxygen species (ROS). CTR indicates not silenced cells (cells incubated with lipofectamine + scramble siRNA). Values are expressed as mean ± SEM (N = 3–4) *p < 0.05; **p < 0.01, ***p < 0.001 obtained by using the student T test

We investigated the biological effects of the reduced Ndufc2 expression in cardiomyocytes. We observed the increase of cell size in *Ndufc2* silenced H9c2 cells (*Ndufc2*-KD) (Fig. 1A), with the parallel up-regulation of *Nppa* and β-*mhc* mRNA levels, known markers of hypertrophy (Fig. 1B). These results demonstrate that the *Ndufc2* reduction leads to cardiomyocytes hypertrophy.

We previously showed that *Ndufc2* knockdown impairs mitochondrial function in vascular cells, due to the alteration of redox status and the unbalance between the oxidized form of nicotinamide adenine nucleotide (NAD^+^) and its reduced form (NADH) (Forte et al. 2020). Here, we found a decrease of NAD^+^: NADH ratio in *Ndufc2*-KD (Fig. 1C), because of an altered function of mitochondrial CI. Preclinical evidence demonstrated that the decrease of NAD^+^ associated with the development of cardiac hypertrophy (Pillai et al. 2010a, b). NAD^+^ is also a substrate for Sirtuin deacetylases and the decreased SIRT3 activity leads to cardiac hypertrophy in mice (Pillai et al. 2010a, b). We observed a reduced level of SIRT3 in *Ndufc2*-KD (Fig. 1D). SIRT3 was reported to prevent cardiac hypertrophy by activating AMPK. The latter in turn inhibits hypertrophic signals by acting as a negative regulator of AKT. SIRT3 also reduces the oxidative stress via the up-regulation of antioxidant system, such as MnSOD (Pillai et al. 2010a, b). We found a decreased activation of AMPK (Fig. 1E), a parallel increase of phosphoAKT (Fig. 1F), a reduced level of MnSOD (Fig. 1G) in *Ndufc2*-KD. We also found that *Ndufc2* silencing increased total reactive oxygen species (ROS) (Fig. 1H). These results suggest that the decrease of NAD^+^ in *Ndufc2*-KD triggers oxidative stress and cell hypertrophy through the modulation of SIRT3 downstream effectors.

#### NMN rescues cell hypertrophy in Ndufc2-silenced CMs

**Fig. 2:**
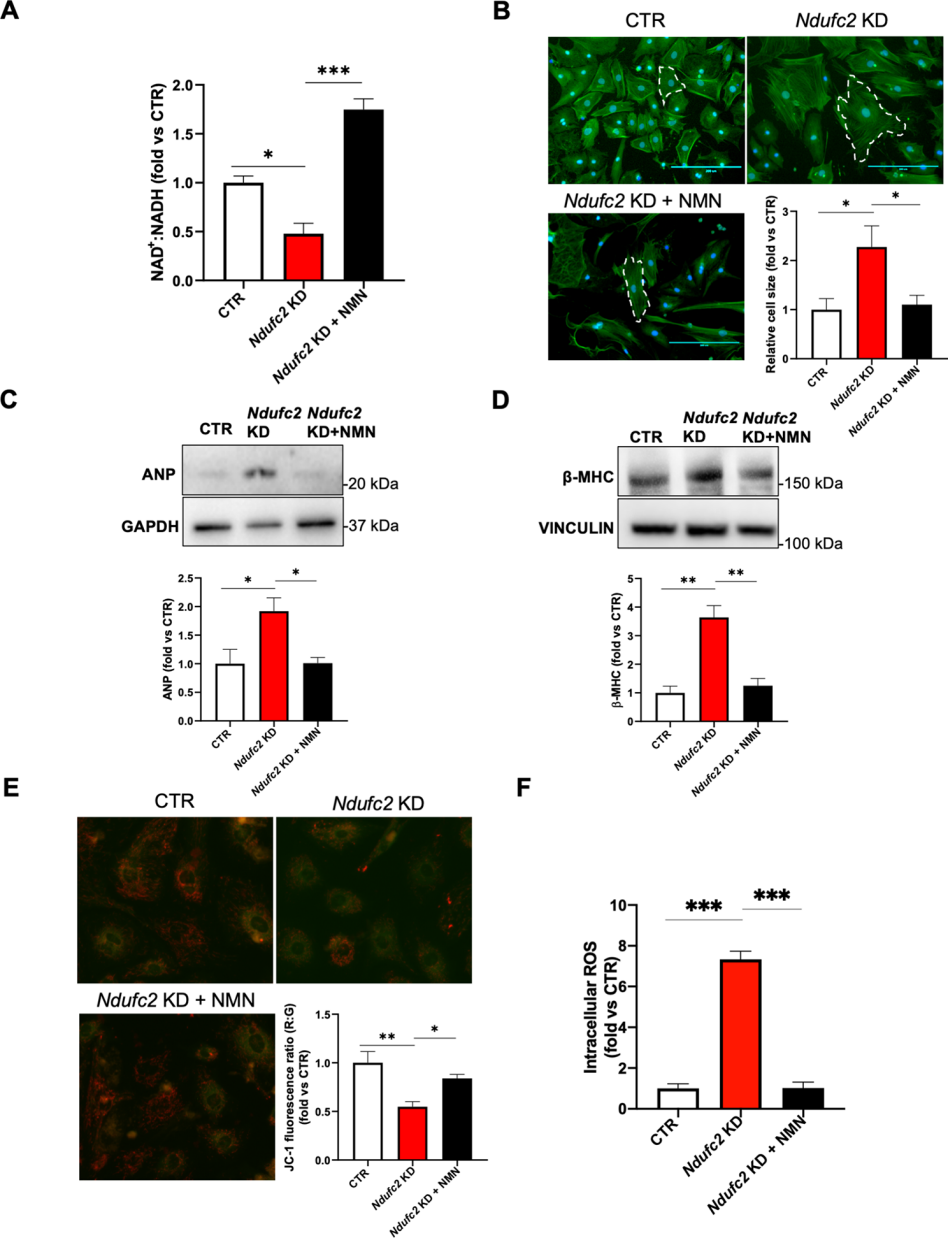
Nicotinamide mononucleotide (NMN) rescues cell hypertrophy and mitochondrial dysfunction in *Ndufc2*-gene silenced primary neonatal rat CMs. CMs were silenced for Ndufc2 (*Ndufc2*-KD) for 48 h. **(A)** NAD+: NADH ratio. **(B)** Representative images of β-actin fluorescence and relative quantification are shown. **C-D.** Representative western blot and corresponding quantification for ANP and β-MHC. **E.** Representative images of JC-1 and corresponding quantification of the red:green ratio (R:G). **F.** Determination of intracellular reactive oxygen species (ROS). NMN was administered to CMs for 48 h at 1 µM. CTR indicates not silenced cells (cells incubated with lipofectamine + scramble siRNA). Values are expressed as mean ± SEM (N = 3–4) *p < 0.05; **p < 0.01, ***p < 0.001 obtained by using the one-way ANOVA followed by Bonferroni’s Multiple Comparison Test

Next, we performed rescue experiments in *Ndufc2*-silenced primary cardiomyocytes (CMs) in the presence of NAD^+^ supplementation through NMN (Fig. 2A). First, we confirmed that *Ndufc2* silencing induced CM hypertrophy, as in H9c2 cells. Then, we found that NMN reduced cell hypertrophy (Fig. 2B-D) in *Ndufc2*-silenced CMs, as observed by the reduction of CM cell size (Fig. 2B) and the decreased ANP and β-MHC levels (Fig. 2C,D), compared to *Ndufc2-*silenced CMs not receiving NMN treatment. We observed that the reduction of cell hypertrophy in cells treated with NMN was associated with an overall amelioration of mitochondrial function and of oxidative status. In fact, we observed the improvement of mitochondrial membrane potential (Fig. 2E) and a reduction of intracellular ROS (Fig. 2F). These results indicate that NMN may represent a valid option to reduce LVH, as suggested by others (Pillai et al. 2010a, b).

#### NDUFC2 and SIRT3 levels decrease in PMBCs of individuals carrying the TT/rs11237379 genotype

**Fig. 3:**
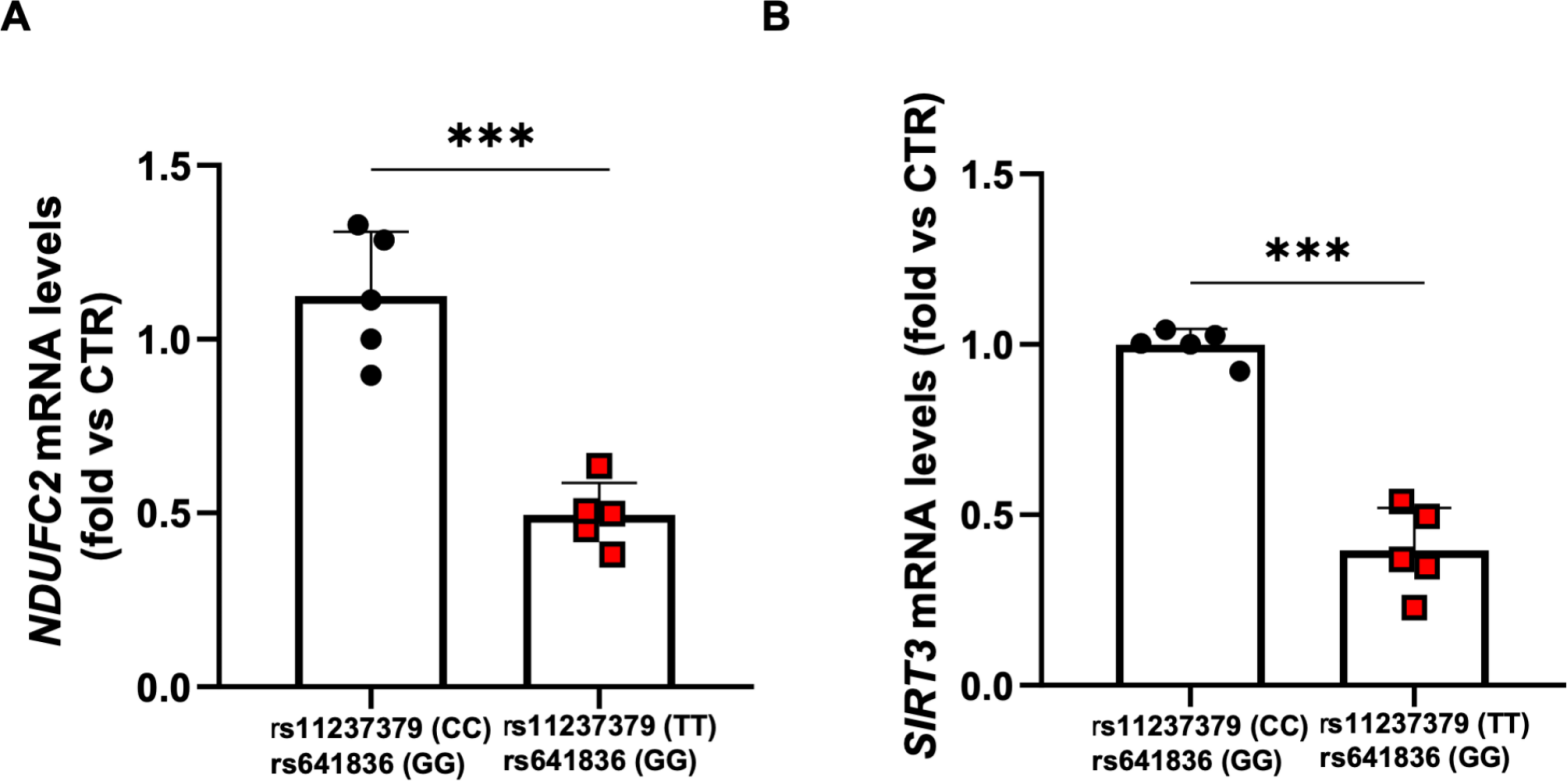
Expression levels of *NDUFC2* and *SIRT3* in PBMCs isolated from hypertensive subjects carrying either TT or CC at rs11237379 and being wild type at rs641836 (GG). **A-B.** Relative quantification by RTqPCR of *NDUFC2* and *SIRT3* in hypertensive subjects carrying the two *NDUFC2*/rs11237379 genotypes. Values are expressed as mean ± SEM (N = 5) ***p < 0.01, obtained by using the student T test

To translate our findings to humans, we assessed the mRNA levels of both *NDUFC2* and *SIRT3* in PBMCs isolated from hypertensive subjects carrying either TT or CC genotype at rs11237379 and being wild type at rs641836. We confirmed a reduced expression of *NDUFC2* in hypertensive patients carrying the TT/rs11237379 genotype, as previously reported. Moreover, hypertensive patients carrying the TT genotype had a reduced *SIRT3* expression (Fig. 3A-B).

## Discussion

Our original data demonstrate that two variants at *NDUFC2* are significantly associated with the presence of LVH in a cohort of Italian hypertensive patients. In the specific, carrier status of either the A allele at *NDUFC2*/rs641836 or the TT genotype at *NDUFC2/*rs11237379 was associated with significantly increased cardiac wall thickness and LV mass independently from BP levels as well as from anthropometric, clinical, and pharmacological parameters. The combined presence of A allele at rs641836 and TT genotype at rs11237379 associated with increased LVH.

Of note, the rs11237379 is known to associate with a significant downregulation of *NDUFC2* expression and consequent CI dysfunction with increased oxidative stress (Raffa et al. 2017; Rubattu et al. 2016). No evidence of a functional role of rs641836 is available yet. The two variants are not in linkage disequilibrium.

The in vitro studies, performed to isolate the impact of reduced *NDUFC2* expression in cardiomyocytes, underscored a significant role of Ndufc2-CI-dependent mitochondrial dysfunction and oxidative stress on cell hypertrophy development and highlighted SIRT3 as a major underlying signaling pathway.

The issue of a cause-effect relationship of mitochondrial dysfunction in cardiac hypertrophy is still debated. The mitochondria structural and functional impairment detected in hypertrophied hearts could be the consequence rather than the cause of hypertrophy. Few studies were previously conducted in the SHR model to clarify this issue. The mitochondria of SHR with compensatory ventricular hypertrophy exhibited impaired enzyme activities of ETC complexes and decreased protein level of some complex subunits, also associated with a dysfunction of the SIRT1/AMPK-Peroxisome proliferator-activated receptor-gamma coactivator (PGC)-1α axis. In this experimental context, a dysregulation of mitochondrial dynamic and mitophagy was detected resulting in a clustered mitochondrial formation as well as increased average size of mitochondria in cardiomyocytes (Tang et al. 2014). Another study found significant alterations of mitochondrial protein spots in the hypertrophied ventricle of SHR. The trifunctional enzyme alpha subunit (Hadha) and the CI Ndufa10 subunit showed modifications of their expression pattern (Meng et al. 2009). This evidence supported the existence of an association of mitochondrial dysfunction with cardiac hypertrophy in hypertension, but it did not clarify the issue of a cause/effect relationship.

A direct role of CI-dependent mitochondrial dysfunction in cardiac hypertrophy development has been revealed by both in vitro and in vivo experimental studies. Indeed, in vitro mechanistic studies showed that *Ndusf1-*KO decreased mitochondrial DNA content, increased mitochondrial ROS production, and caused cardiomyocyte hypertrophy with enhanced expression of ANP, brain natriuretic peptide (BNP), and β-MHC. Furthermore, the expression level of *Ndusf1* mRNA and protein was downregulated in hypertrophied cardiac tissue, supporting the important role of *Ndusf1* deletion and CI dysfunction in the development and progression of cardiac hypertrophy (Ni et al. 2019; Zou et al. 2021). Another study demonstrated that *Ndufs4*-null mice had a decrease of about 50% in cardiac CI activity and developed severe hypertrophic cardiomyopathy as assessed by magnetic resonance imaging (Chouchani et al. 2014).

Our findings obtained with the Ndufc2 subunit, while reinforcing the previous experimental results obtained with other CI subunits, provide the first evidence of a significant association between a mechanism underlying CI-dependent mitochondrial dysfunction and cardiac hypertrophy in human hypertension. The subsequent molecular investigation by knocking-out *Ndufc2* in isolated cardiomyocytes added important mechanistic insights to the associative evidence obtained in the human sample, proving a direct cause-effect relationship between Ndufc2-dependent CI dysfunction and the development of cardiomyocytes hypertrophy.

As previously shown, Ndufc2 is a fundamental component of NADH dehydrogenase to allow regular CI assembly and activity (Rubattu et al. 2016). Indeed, Ndufc2 deficiency causes decreased CI integrity and function, decreased mitochondrial membrane potential and ATP production, enhanced ROS accumulation and inflammation. Along with the functional impairment, a remarkable ultrastructural damage of mitochondria is produced (Raffa et al. 2017; Rubattu et al. 2016). Consequently, cell viability decreased whereas necrosis increased (Raffa et al. 2017). The impact of a Ndufc2 dysregulation in human diseases has been so far documented by its relationship with an increased susceptibility to cardiovascular events (Gallo et al. 2022; Rubattu et al. 2016).

Consistently with previous studies on different CI subunits, our *in-vitro* studies showed that *Ndufc2*-silenced cardiomyocytes had a significant degree of hypertrophy that was associated with an increased expression level of both ANP and β-MHC. Exposure to NMN, a strategy able to rescue CI function (19), reduced cellular volume and gene expression level of both hypertrophy markers. Of note, we found a reduced expression of SIRT3. These results were obtained in both commercially available and rat primary cardiomyocyte cell lines. Further investigation of the downstream molecules expression in H9c2 cells revealed a decrease of MnSOD and phospho-AMPK with a parallel increase of phospho-AKT in *Ndufc2*-KD.

SIRT3 plays a protective role against mitochondrial damage via regulating the activation of energy metabolism. It modulates AMPK to protect cardiac function during stress conditions, limiting cellular damage through the modulation of mitochondrial respiration (Kane & Sinclair 2018, Zhang et al. 1999). SIRT3 has been previously demonstrated to counteract cardiac hypertrophy in primary cultures of cardiomyocytes by activating MnSOD and catalase, thereby decreasing cellular levels of ROS. In turn, reduced ROS levels suppressed Ras activation and the downstream signaling pathway through the MAPK/ERK and PI3K/Akt pathways. Finally, this phenomenon repressed the activity of both transcription factors, specifically GATA4 and NFAT, and translation factors, namely eukaryotic initiation factor 4E (elf4E) and ribosomal protein S6 kinase (PS6K), which are involved in the development of cardiac hypertrophy (Sundaresan et al. 2009). Therefore, a reduced expression of *SIRT3*, such as that observed in *Ndufc2*-silenced cardiomyocytes, is a plausible mechanism contributing to cellular hypertrophy. Interestingly, the in vitro evidence was paralleled by the observation that, in addition with *NDUFC2* expression, *SIRT3* expression was markedly downregulated in PBMCs of human patients carrying the TT genotype at rs23117379. Therefore, our data provide further evidence of a biological link underlying the association between *NDUFC2*, *SIRT3* and cardiac hypertrophy.

Our study also presents some limitations. We included only Caucasian patients from a single center, thus limiting the possibility to extend our results to other ethnic groups. Additional studies in different cohorts are required to reinforce the current evidence on the contributory role of Ndufc2 deficiency to LVH development in human hypertension.

It is very likely that *NDUFC2*/rs641836 also associates with reduced gene expression. Although we attempted to gain some insights on the biological relevance of this variant, the difficulty in isolating subjects carrying the mutant allele at this gene variant, even in heterozygosity, while being wild type at rs11237379 hampered our efforts.

## Conclusions

We demonstrate that two variants at *NDUFC2* are significantly associated with the presence of cardiac hypertrophy in a cohort of Italian hypertensive patients. The T allele at rs11237379 variant is known to associate with reduced gene expression and mitochondrial dysfunction. The in vitro studies, performed to isolate the impact of reduced *Ndufc2* expression in cardiomyocytes, revealed a significant role of CI-dependent mitochondrial dysfunction on cell hypertrophy development and highlighted SIRT3 as a major underlying signaling pathway.

These findings provide significant evidence of a direct contributory role of Ndufc2 dysregulation in cardiomyocytes hypertrophy development in vitro and that the consequent CI dependent mitochondrial dysfunction plays an important role in LVH development in human hypertension. Through the analysis of Ndufc2, we extend previous experimental evidence obtained with other CI subunits. To our knowledge, these results represent the first translation of experimental findings to humans regarding the role of CI-dependent mitochondrial dysfunction in cardiac hypertrophy development. Moreover, these results highlight a major role of the SIRT3 pathway as a mechanism mediating the effect of *Ndufc2* deletion in cardiomyocytes. These emerging data have obvious and non-negligible theoretical and applicative implications. Our results pave a way of a new pathophysiological mechanism of LVH in humans as potential therapeutic target in subjects at high risk to develop this pathology which may lead to new diagnostic, clinical and therapeutic strategies.

### Electronic supplementary material

Below is the link to the electronic supplementary material.

Supplementary Material 1

## Data Availability

The data during the current study are available from the corresponding author on reasonable request.

## Abbreviations

CI: Complex I
ETC: Electron transport chain
LVH: Left ventricular hypertrophy
NMN: Nicotinamide mononucleotide
PBMCs: Peripheral blood mononuclear cells
SBP: Systolic blood pressure
SIRT3: Sirtuin 3
