# Supplementary material for: Polymorphic variants at NDUFC2, encoding a mitochondrial complex I subunit, associate with cardiac hypertrophy in human hypertension
Source: Mol Med. 2023 Aug 9;29:107. doi: 10.1186/s10020-023-00701-x (PMC10410816; doi:10.1186/s10020-023-00701-x)
Supplement: Supplementary file 1 — Supplementary Material 1 [file 10020_2023_701_MOESM1_ESM.docx]

**ADDITIONAL FILE 1**

**Polymorphic variants at *NDUFC2*, encoding a mitochondrial complex I subunit, associate with cardiac hypertrophy in human hypertension**

Gallo et al.


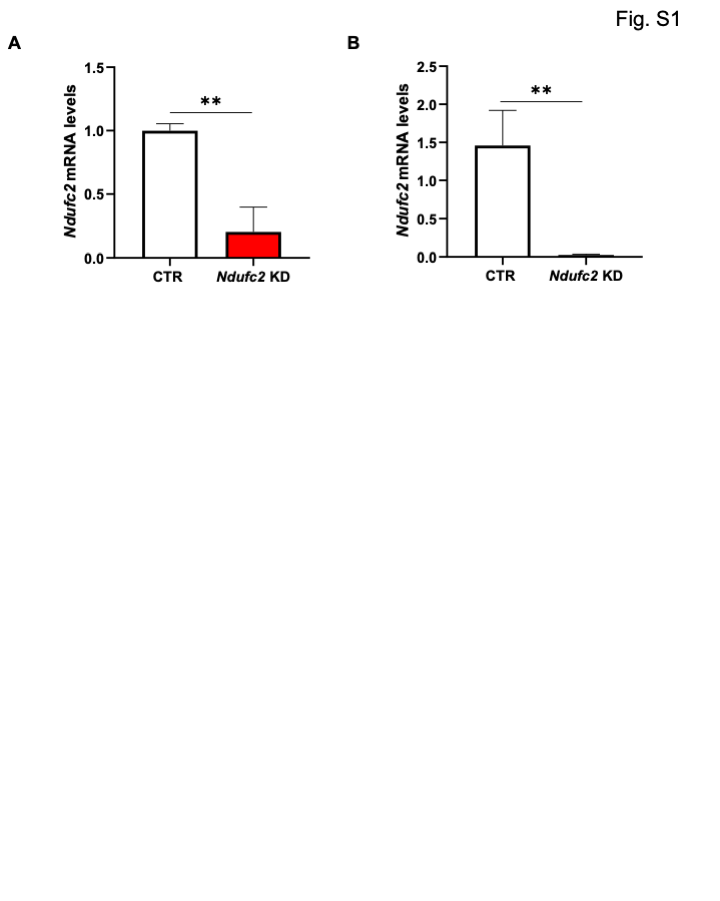


**Fig. S1** Efficacy of *in-vitro* gene silencing of *Ndufc2*. **A-B.** Relative quantification by RTqPCR of NDUFC2 in H9c2 cells (**A**) and in primary neonatal rat cardiomyocytes (CMs) (**B**). Values are expressed as mean ± SEM (N=3-4). CTR indicates not silenced cells (cells incubated with lipofectamine + scramble siRNA) **p<0.01, obtained by using the student T test.
